# Role of GeneXpert MTB/Rif Assay in Diagnosing Tuberculosis in Pregnancy and Puerperium

**DOI:** 10.1155/2015/794109

**Published:** 2015-08-03

**Authors:** Zaiyad G. Habib, Farouq M. Dayyab, Abdallah Sanda, Sirajo H. Tambuwal, Mahmood M. Dalhat, Hamza Muhammad, Garba Iliyasu, Ibrahim Nashabaru, Abdulrazaq G. Habib

**Affiliations:** ^1^Infectious & Tropical Diseases Unit, Department of Medicine, Aminu Kano Teaching Hospital, PMB 3452, Kano 700233, Kano State, Nigeria; ^2^Department of Medical Microbiology, Aminu Kano Teaching Hospital, PMB 3452, Kano 700233, Kano State, Nigeria; ^3^College of Health Sciences, Bayero University, Kano, PMB 3011, Kano 700241, Kano State, Nigeria

## Abstract

Presentation of tuberculosis (TB) in pregnancy may be atypical with diagnostic challenges. Two patients with complicated pregnancy outcomes, foetal loss and live premature delivery at 5 and 7 months of gestation, respectively, and maternal loss, were diagnosed with pulmonary TB. Chest radiography and computed tomography showed widespread reticuloalveolar infiltrates and consolidation with cavitations, respectively. Both patients were Human Immunodeficiency Virus (HIV) seronegative and sputum smear negative for TB. Sputum GeneXpert MTB/Rif (Xpert MTB/RIF) was positive for *Mycobacterium tuberculosis*. To strengthen maternal and childhood TB control, screening with same-day point-of-care Xpert MTB/RIF is advocated among both HIV positive pregnant women and symptomatic HIV negative pregnant women during antenatal care in pregnancy and at puerperium.

## 1. Introduction

Tuberculosis (TB) diagnosis may be challenging among those with immunosuppressive conditions like Human Immunodeficiency Virus (HIV) infection and pregnancy [1, 2]. Such patients are often anergic with negative tuberculin skin test, low sputum smear yield for acid fast bacilli (AFB), atypical presentations, and pregnancy related weight gain that masks TB wasting [1, 2]. Active TB is occasionally encountered among pregnant women in endemic countries or among migrant population in nonendemic countries. Given the challenges in diagnosis, the time-limited nature of pregnancy, and the caution desired prior to exposure to X-rays in conducting chest radiography, newer tools that are reliable, simple, and proven in immunosuppressive conditions and have rapid turnaround time are needed to optimize detection of TB in pregnancy. GeneXpert MTB/Rif assay (Xpert MTB/RIF) has been used to diagnose TB in HIV infected patients and in sputum smear negative patients [3, 4]. The World Health Organization (WHO) guideline gave strong and conditional recommendations for use of Xpert MTB/RIF. The guideline strongly recommended use of Xpert MTB/RIF as the initial diagnostic test in adults and children presumed to have MDR-TB or HIV-associated TB and as the initial diagnostic test in testing cerebrospinal fluid specimens from patients presumed to have TB meningitis. The guideline also gave conditional recommendations in the presence of major resource implications. This conditional recommendation includes using Xpert MTB/RIF as the initial diagnostic test in adults and children presumed to have TB and as a follow-on test to microscopy in adults presumed to have TB but not at risk of MDR-TB or HIV-associated TB especially in further testing of smear negative specimens. Xpert MTB/RIF can also be used conditionally as a replacement test for usual practice (including conventional microscopy, culture, and/or histopathology) for testing of specific nonrespiratory specimens (lymph nodes and other tissues) from patients presumed to have extrapulmonary TB [5]. In a study in Zambia among inpatients with a primary obstetrics or gynaecological problem that had concomitant cough, Xpert MTB/RIF when compared to standard sputum smear microscopy, MTB culture, and MTB drug susceptibility testing provided a reasonably sensitive, specific, and rapid method for diagnosing pulmonary TB in obstetrics and gynaecological patients. Out of the 27.7% of culture confirmed cases of pulmonary TB from the study, Xpert MTB/RIF assay had a sensitivity of 80.8% compared with MTB culture while sputum smear microscopy had a sensitivity of 50.0% compared with the MTB culture [6]. In our study, we described the use of Xpert MTB/RIF in diagnosing pulmonary TB in patients with complicated pregnancy and puerperium and discuss the implications for maternal and child health.

## 2. Case 1

### 2.1. Day 0 of Admission

The first case was that of a 24-year-old booked primigravida who presented a few days following abortion at 5 months of gestation to the infectious diseases clinic with breathlessness, cough, and fever of 2-week duration following evacuation for incomplete abortion. There was history of weight loss and haemoptysis. When examined she was found to be pale and febrile (Temperature = 39.5°C axillary). She was tachycardic with a pulse rate of 120 beats per minute; blood pressure was 90/70 mmHg and she had normal heart sounds. She was also tachypnoeic (respiratory rate: 32 cycles per minute) with oxygen saturation of 70% on room air. She had coarse crackles and bronchial breathing. Differentials considered were severe community acquired pneumonia, sepsis secondary to pelvic infection, and pulmonary embolism secondary to pelvic inflammatory disease with pelvic thromboembolism.

Investigations requested include full blood count (FBC), erythrocyte sedimentation rate (ESR), chest radiograph, sputum microscopy and culture for bacteria, sputum for fungal study, sputum smear and microscopy for acid fast bacilli, blood culture, Doppler ultrasound of the pelvis and lower limbs, electrocardiogram (ECG), urea and electrolytes, urine microscopy, culture and sensitivity, urinary pregnancy test, and HIV screening.

She was commenced on initial empiric therapy of piperacillin-tazobactam, doxycycline, gentamicin, intravenous fluids, and oxygen supplementation. Vancomycin and enoxaparin were subsequently added.

### 2.2. Initial Results Obtained on Day of Admission

**Figure 1:**
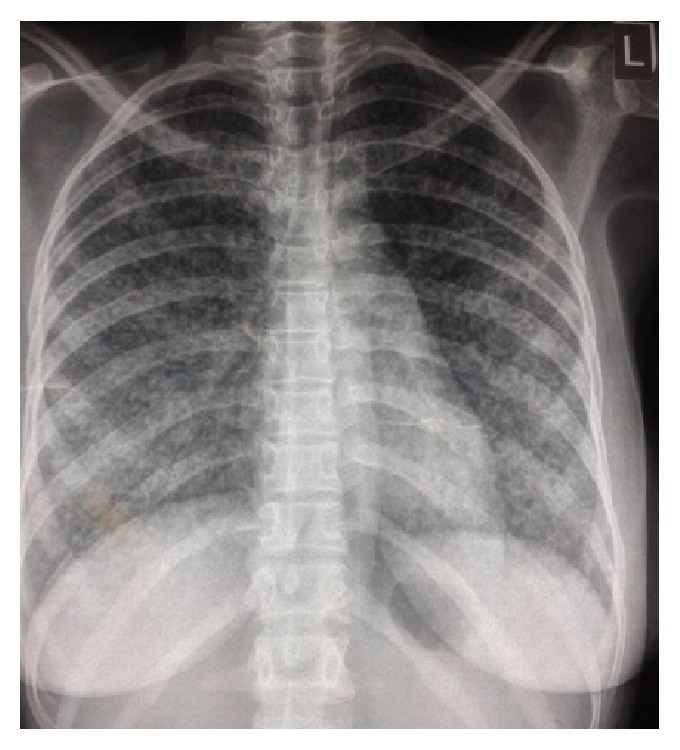
Chest radiograph showing widespread (whole lung) reticuloalveolar “miliary” infiltrates.

Electrocardiography (ECG) showed sinus tachycardia with no diagnostic features of pulmonary embolism and screening for HIV was negative. Initial full blood counts (FBC) showed white blood cell counts (WBC) of 9.7 × 10^9^/L, neutrophils of 84.6%, lymphocytes of 11.9%, haematocrit (HCT) of 37%, and platelets of 273 × 10^9^/L. Preliminary sputum Gram stain showed Gram negative bacilli and Gram positive cocci. Doppler ultrasound scan of the lower limbs was negative for deep venous thrombosis of calf veins. Chest radiograph showed global infiltrates reported by radiologist as reticuloalveolar “miliary” shadows (Figure 1).

### 2.3. Day 1 of Admission

Patient was still febrile (Temperature = 38.0°C axillary). Her vital signs showed respiratory rate of 48 cycles/minute, pulse rate of 140, and blood pressure of 86/60 mmHg. She was still on oxygen supplementation and pulse oximetry (SpO_2_) was 93%.

Three sputum smears for acid fast bacilli (AFB) were negative. Urinary pregnancy test also came back negative. GeneXpert MTB/Rif was ordered.

Her treatment was continued and doxycycline was discontinued.

### 2.4. Day 2 of Admission

Patient was still febrile, tachypnoeic, tachycardic, and hypotensive.

Sputum microscopy and culture for fungal pathogens were negative. Microscopy for bacteria showed Gram positive diplococci and Gram negative bacilli and* Streptococcus pneumoniae* was cultured from the sputum. Piperacillin-tazobactam was substituted with levofloxacin based on sensitivity results. Automated blood cultures yielded no growth.

GeneXpert MTB/Rif version 5.0 Cepheid assay (Xpert MTB/RIF) was positive for* Mycobacterium tuberculosis* with no detectable rifampicin resistance. Patient remained pyrexic and tachypnoeic for 3 days despite antibiotics. She was then commenced on four-drug anti-TB therapy (rifampicin, isoniazid, pyrazinamide, and ethambutol).

### 2.5. Day 4 of Admission

Two days after commencing anti-TB therapy, patient was less dyspnoeic and afebrile (Temperature = 37.1°C axillary); SPO_2_ rose to 89% on room air and 98% on oxygen supplementation. Though she was still tachycardic with a radial pulse rate of 128 beats per minute, her blood pressure rose to 100/70 mmHg.

FBC done showed WBC of 8.1 × 10^9^/L, neutrophils of 74.9%, lymphocytes of 14.5%, HCT of 32.3%, platelets of 307 × 10^9^/L, and erythrocyte sedimentation rate (ESR) of 20 mm/hr.

### 2.6. Day 5 of Admission

Patient suddenly desaturated in the evening with breathlessness and shock and subsequently died. An autopsy could not be performed on the patient due to the religious belief of the patient as she was a Muslim and it was required that she should be buried immediately upon death.

## 3. Case 2

### 3.1. Day 0 of Admission

A 27-year-old primipara presented 2 weeks after a spontaneous vaginal delivery of a live baby with a 6-week history of productive cough, associated right sided pleuritic chest pain with no haemoptysis. The cough became exacerbated following delivery. There were low grade intermittent fever and drenching night sweats with initial onset of symptoms 6 weeks to presentation. The pregnancy was booked. She had a live preterm delivery at 7 months of gestation. At presentation she was febrile (Temperature = 38.8°C axillary), had left supraclavicular lymphadenopathy, tachypnoea (28 cycles per minute), and bibasal crackles, and was also tachycardic (146 beats per minute). Differentials considered were community acquired pneumonia, sepsis, pulmonary embolism, and pulmonary tuberculosis.

Investigations requested include full blood count (FBC), erythrocyte sedimentation rate (ESR), chest radiograph, sputum microscopy and culture for bacteria, sputum for fungal study, sputum smear and microscopy for acid fast bacilli, blood culture, pelvic ultrasound, electrocardiogram (ECG), urea and electrolytes, urine microscopy, culture and sensitivity, high vaginal swab (HVS) culture, and HIV screening. The patient was commenced on empiric ceftriaxone, ciprofloxacin, enoxaparin, and an intravenous infusion and also had a stat dose of vancomycin.

### 3.2. Day 1 of Admission

Patient was still febrile, tachypnoeic, and tachycardic.

Her initial results showed WBC of 4.8 × 10^9^/L, haemoglobin of 11.8 g/L, platelets of 164 × 10^9^/L, and ESR of 130 mm/hr, with normal electrolytes and liver function tests. Chest X-ray showed streaky opacities in the left mid and lower zones. Preliminary sputum smear showed Gram positive cocci in chains and Gram negative rods. Pelvic ultrasound showed retained products. ECG showed sinus tachycardia. Viral screening for HIV, hepatitis B, and hepatitis C was negative.

Clindamycin was added to her treatment as well as a stat dose of gentamicin. Ceftriaxone and vancomycin were however stopped to continue with ciprofloxacin.

Patient also had manual vacuum aspiration (MVA) done where about 80 mL of products was removed.

### 3.3. Day 2 of Admission

Patient was still febrile, tachypnoeic, and tachycardic and still complaining of right sided pleuritic chest pain.

### 3.4. Day 5 of Admission

Patient was still febrile; sputum culture revealed ESBL* Klebsiella pneumoniae* sensitive to only imipenem and meropenem; hence meropenem was commenced. All other cultures yielded no growth. Three sputum Ziehl-Neelsen stains were also negative.

### 3.5. Day 8 of Admission

The patient was still febrile (Temperature = 39.5°C axillary) and coughing and still having tachycardia.

Further investigations were done with the following results: 2D-echocardiogram showed pericarditis with minimal effusion. She had serum D-dimer of 734.46 ng/mL (0–500 ng/mL), negative serum antinuclear antibody (ANA).

**Figure 2:**
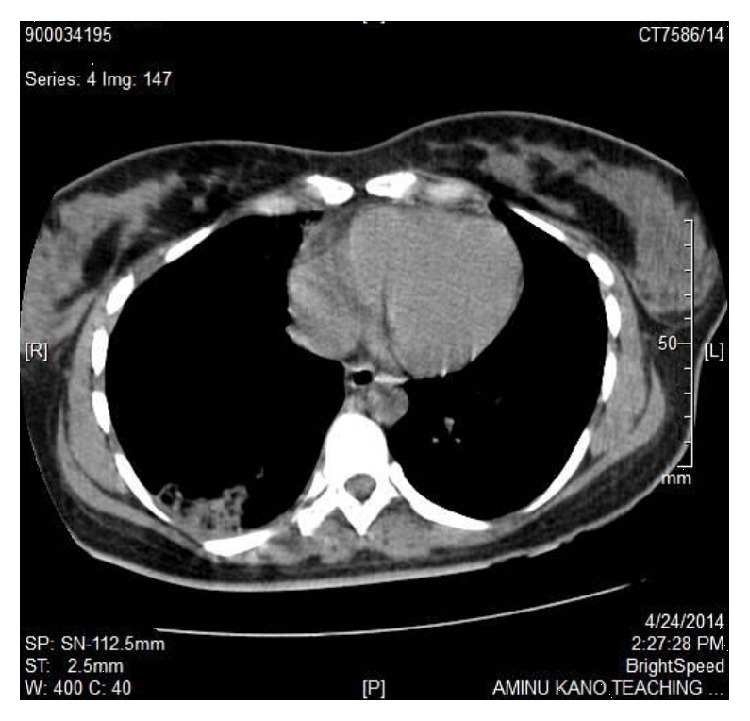
Chest CT scan showing consolidation with “cavities” in the right posterior lung base.

Spiral chest CT Scan excluded pulmonary arterial embolism but showed a well-defined fairly rectangular area of parenchymal density (HU: 11–36) that had scattered areas of hypoattenuation “cavitations” within it, seen in the posterior aspect of the right basal lung zone (Figure 2).

**Figure 3:**
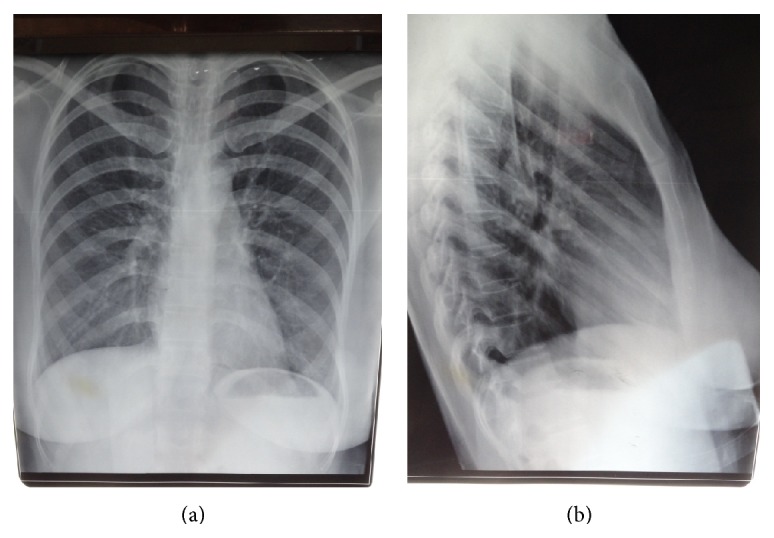
Posteroanterior and lateral chest radiograph, respectively, showing clear lung fields after completion of six-month antituberculous medication.

Xpert MTB/RIF done detected* Mycobacterium tuberculosis* with no rifampicin resistance. A diagnosis of disseminated TB involving lungs, lymph nodes, and pericardium was considered. The patient received four-drug antituberculous medication (rifampicin, isoniazid, ethambutol, and pyrazinamide) with dramatic defervescence after 4 days and noticeable gradual improvement. Ethambutol was later stopped when the patient developed visual side effects 7 days after its commencement. Follow-up of patient at just four weeks of treatment showed WBC of 3.4 × 10^9^/L, haemoglobin of 11.8 g/L, platelets of 352 × 109/L, and ESR of 55 mm/hr. Her serum electrolytes and liver function tests remained normal. She was discharged and had complete six months of antituberculous medication. Posteroanterior and lateral chest radiographs at completion of medication showed clear lung fields with complete resolution of the lesion on the posterior aspect of the right basal lung zone (Figures 3(a) and 3(b), resp.).

## 4. Discussion

The cases presented described the diagnosis of TB by GeneXpert MTB/Rif assay (Xpert MTB/RIF) in two women who are HIV negative following abortion and live preterm delivery, respectively. The first patient had a chest radiograph consistent with miliary TB while the second patient had nonspecific chest radiograph changes with chest CT scan however having features suggestive of pulmonary TB. Sputum smear was negative for AFB in both cases. The second patient had markedly elevated ESR. Doppler ultrasound, ECG, and chest spiral computed tomography excluded deep venous thrombosis and pulmonary embolism. The patients were treated with antibiotics for presumed sepsis (community acquired pneumonia and pelvic inflammatory disease) with no response.* Streptococcus pneumoniae* and* Klebsiella pneumoniae* were, respectively, cultured in the sputum of the cases and were likely super added acute bacterial infections. However, the symptoms and radiographic lesions actually persisted or worsened despite follow-up culture after antibiotic course yielding no growth. Subsequently, commencement of anti-TB therapy led to immediate resolution of symptoms with gradual improvement of the second patient but unfortunately led to worsening of symptoms and a fatal outcome in the first patient which likely could have been from overwhelming TB or immune reconstitution reaction.

Diagnosis of TB in immunocompromised states like HIV and pregnancy could be challenging as sputum smear AFB yield is often poor [1, 2]. Indeed in a study of 602 HIV-associated TB patients, sputum smear had a low sensitivity of 26.7% compared to Xpert MTB/RIF with >80%. It remained sensitive even among those with severe or advanced disease with low CD4 counts of <100 cells/*μ*L while only 15% were culture positive [3]. Similarly, in a study conducted among obstetrics and gynaecology patients, a sensitivity of 80.8% from Xpert MTB/RIF compared to about 50% from sputum smear was reported; hence as an alternative to sputum microscopy, Xpert MTB/RIF provides a sensitive, specific, and rapid method for the diagnosis of pulmonary TB among obstetric and gynaecological patients [6]. Although our patients were HIV seronegative and had chest radiograph and chest CT scan suggestive of pulmonary TB, respectively, immunosuppressed patients from any cause including pregnancy often present with atypical chest radiographs. Lesions are found in unusual locations as in the second case and Xpert MTB/RIF in this instance provides a more accurate TB diagnosis than chest radiography [7].

As typified by our patients who had radioimaging only after delivery, clinicians often are cautious in conducting radiography and computed tomography during pregnancy due to concerns of potential untoward effects of exposure to radiation to the foetus. This further precludes the opportunity to detect TB timely during pregnancy and can lead to either premature delivery, foetal loss, and/or maternal loss as seen in the two cases.

Indeed, diagnosis of TB and commencement of therapy were delayed in the first case that had pregnancy loss and eventually proved fatal to the mother. Even though symptoms preceded delivery by a prolonged period, diagnosis in the second patient was also delayed until puerperium with potential risk of vertical transmission to the baby. However after thorough evaluation there was no evidence of vertical transmission of TB to the baby in the second case. Compared to sputum smear, Xpert MTB/RIF has been shown to have a higher sensitivity to detect TB much more rapidly in sputum smear negative patients and in such cases where culture has to be relied upon, Xpert MTB/RIF reduces the median time of making a diagnosis of TB by 29 days among suspected patients [8]. Reasonably accurate and reliable results may be available within hours or within a day with Xpert MTB/RIF. Potentially smear microscopy combined with Xpert MTB/RIF provides an opportunity for strengthening TB screening during ANC among both symptomatic and asymptomatic pregnant women. At population level the combination of the two approaches has been found to be complementary and synergistic [9]. A potential platform at the ANC will be to situate the two tests in the context of Prevention of Mother-to-Child Transmission (PMTCT) of HIV infection [6, 9, 10]. If logistics are properly managed the assay can be conducted as a point-of-care same-day test and therapy commenced for those confirmed positive. Already such ANC point-of-care same-day rapid HIV tests are offered. A similar immunochromatographic syphilis test has been introduced and its scale-up in Sub-Saharan Africa is being explored for the control of maternal and congenital syphilis [11]. Both cases occurred after delivery or in puerperium and a recent large study showed that early postpartum women are twice as likely to develop TB as nonpregnant women [2]. Therefore, screening for TB may need to be continued during puerperium.

Automated liquid or solid TB culture and drug susceptibility testing will confirm the diagnosis but take a much longer period of at least about 2 or 6–8 weeks, respectively.

Xpert MTB/RIF can also detect rifampicin resistance, a predictor of multidrug resistant TB. As many of the second-line drugs are relatively toxic and unsafe in pregnancy, information on drug resistance will be crucial in guiding selection of appropriate second-line anti-TB medications and facilitating optimum management of mother and child.

Even in Sub-Saharan Africa with a low ANC coverage, 23.5 million (74%) of 32 million annual live births were from women reported to have had at least one ANC visit [11]; point-of-care same-day ANC Xpert MTB/RIF has the potential to reduce mother-to-child transmission of* Mycobacterium tuberculosis* while strengthening existing TB control in neonatal period as case detection will be conducted more timely. Mothers and babies detected could be commenced on anti-TB therapy promptly and isoniazid preventive therapy may be offered to unaffected children of mothers on treatment followed by BCG vaccination.

In conclusion, two women with poor pregnancy outcomes, foetal loss and live preterm delivery and maternal loss, were diagnosed with pulmonary TB. Maternal mortality resulted in the first patient. Sputum smear was negative and diagnosis of TB was by Xpert MTB/RIF with suggestive chest radiograph and chest CT scan for the first and the second patients, respectively. To strengthen maternal and childhood TB control, screening with same-day point-of-care Xpert MTB/RIF is advocated among symptomatic women during pregnancy and puerperium.
